# Inter-centre reproducibility of cardiac diffusion tensor measures

**DOI:** 10.1186/1532-429X-16-S1-O84

**Published:** 2014-01-16

**Authors:** Elizabeth M Tunnicliffe, Andrew D Scott, Pedro Ferreira, Rina Ariga, Laura-Ann McGill, Sonia Nielles-Vallespin, Stefan Neubauer, Dudley J Pennell, Matthew D Robson, David Firmin

**Affiliations:** 1 OCMR, Radcliffe Department of Medicine, University of Oxford, Oxford, UK; 2 Cardiovascular Biomedical Research Unit, Royal Brompton Hospital, London, UK

## Background

Diffusion tensor imaging is a technique which, by studying the small motions of water molecules in biological tissue, could provide new insights into the myoarchitecture of both healthy and diseased hearts. The apparent diffusion coefficient (ADC) and fractional anisotropy (FA) are values derived from the measured diffusion tensor and which have previously shown some sensitivity to disease. However, previously reported normal values of these parameters have varied widely. The aim of this work was to evaluate the reproducibility of the ADC and FA measured at two separate centres.

## Methods

The stimulated-echo diffusion tensor sequence was as previously described^1^, with a 37.5% field-of-view in the phase-encode direction and fixed monopolar diffusion gradients. This gives b-values proportional to the RR-interval; at 60 bpm b = 15 (reference) and 350 s/mm^2^. These b-value calculations were verified by using the sequence to measure phantoms with known ADC. The sequence was independently implemented at the two centres, a 3T Skyra (Siemens, Erlangen, Germany) at centre A and a TIM Trio (Siemens) at centre B. Each centre implemented separate analysis software in Matlab (Mathworks, Natick, MA). Ten healthy volunteers were scanned at each centre, a maximum of 8 days apart. In each breathhold, one average of a reference image and six diffusion weighted images, with independent diffusion directions, was acquired. Eight averages of a single mid-ventricular short axis slice were acquired. The resulting diffusion images were analysed at both centres to produce ADC and FA maps. Regions of interest (ROIs) covering the left ventricle were manually drawn, and the average ADC and FA calculated, giving four measures per subject (ADC and FA in systole and diastole). Inspection of the data revealed that they were non-normally distributed, so Friedman's repeated measures test as implemented in the R *agricolae *package (R Foundation, Vienna, Austria) was used, with Holm-corrected Fisher's LSD for post hoc testing. The two centres were deemed the same if p > 0.1.

## Results

**Figure 1:**
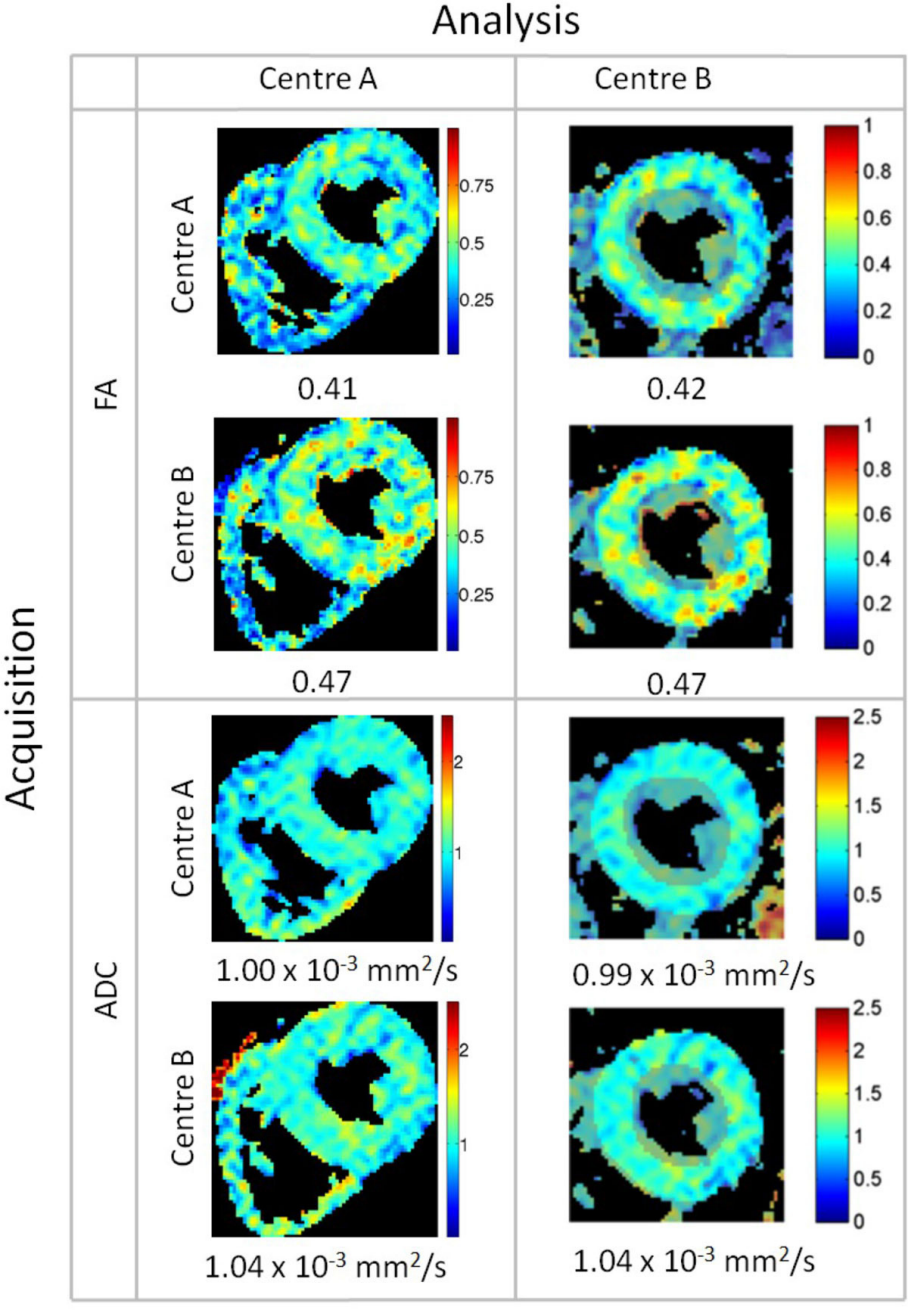
**Examples of FA and ADC maps in systole for a single subject, showing the acquisition and analysis combinations compared in this study**. Beneath each map is the mean, calculated over matched ROIs and shown in the more saturated colour on the maps from Centre B.

Initial results showed statistically significant differences between almost all variables, and the post-hoc analysis indicated that these disparities were driven by differences in the analysis. Therefore the analysis was re-run using only ROIs defined by centre A on all data, the results of which are shown in Table 1. These showed no significant differences between the metrics at the two centres. Example ADC and FA maps for one subject are shown in Figure 1.

**Table 1:** The median measures for data acquired and analysed at the same centre, with p-values for Friedman's test for differences between all acquisition and analysis combinations, showing that with identical ROIs, the differences between the two centres is statistically insignificant.

		Systolic ADC	Systolic FA	Diastolic ADC	Diastolic FA
Median over all 10 subjects ± IQR	Centre A	1.04 ± 0.03 × 10^-3^mm^2^/s	0.41 ± 0.05	1.17 ± 0.14 × 10^-3^mm^2^/s	0.54 ± 0.04
	Centre B	1.15 ± 0.13 × 10^-3^mm^2^/s	0.41 ± 0.05	1.26 ± 0.17 × 10^-3^mm^2^/s	0.55 ± 0.04
	Friedman's test p-value	0.303	0.831	0.273	0.541

## Conclusions

This work demonstrates that, when regions of interest are carefully defined, ADC and FA are reproducible cardiac diffusion measures across different centres, when using a well-matched sequence. However, it may be that interobserver variability is an important factor in the analysis of cardiac diffusion tensor imaging.

## Funding

Parts of this research were funded by the National Institute for Health Research (NIHR) Oxford Biomedical Research Centre based at The Oxford University Hospitals Trust at the University of Oxford, and the NIHR Cardiovascular Biomedical Research Unit at the Royal Brompton Hospital and Imperial College, London. The views expressed are those of the authors and not necessarily those of the NHS, the NIHR or the Department of Health.
